# A Deep Learning-Based Decision Support System for Cholelithiasis in MRI Data

**DOI:** 10.3390/jcm15051891

**Published:** 2026-03-02

**Authors:** Ebru Hasbay, Caglar Cengizler, Mahmut Ucar, Nagihan Durgun, Hayriye Ulkucan Disli, Deniz Bolat

**Affiliations:** 1 Department of Radiology, Izmir City Hospital, Izmir 35530, Turkey; 2 Biomedical Device Technology Program, Vocational School of Health Services, Izmir Democracy University, Izmir 35140, Turkey; caglar.cengizler@idu.edu.tr; 3 Electrical and Electronics Engineering, Izmir Democracy University, Izmir 35140, Turkey; mahmut.ucar@idu.edu.tr; 4 Department of Urology, Izmir City Hospital, Izmir 35530, Turkey

**Keywords:** gallbladder, MRI, deep learning, segmentation, cholelithiasis, R-CNN, Squeeze-and-Excitation, U-Net

## Abstract

**Background:** Cholelithiasis can lead to significant complications if not diagnosed and treated promptly. Recent advances in deep learning and the improved ability of computer systems to detect clinically significant textural and morphological patterns in magnetic resonance imaging (MRI) can help reduce the time and resources required for the radiological evaluation of the gallbladder and cholelithiasis. **Objective:** To detect cholelithiasis, a support system with a graphical user interface for magnetic resonance (MR) images of the gallbladder was implemented to reduce the manual effort and time required to identify gallstones. **Method:** A commonly used deep learning model for pixel-level mask generation and instance segmentation, Mask Region Based Convolutional Neural Network (Mask R-CNN), was modified, trained, and evaluated to provide a robust pipeline for automated analysis. The primary aim was to automatically locate and label the gallbladder in T2-weighted axial MR images to detect gallstones and highlight the visual characteristics of the target region, thereby supporting radiologists. All automation was designed to operate on a single optimal slice instead of the entire volume. While this approach limits generalisability, it offers a practical starting point for method development. This setup reflects a feasibility-oriented design, rather than a comprehensive diagnostic capability. The dataset included 788 axial MR images from different patients. Each image was labeled and segmented by an experienced radiologist to train and test the models at the image level. **Results:** The proposed model with squeeze and excitation (SE) modification improved classification accuracy, and at the image level, stone detection improved in terms of accuracy, precision, and specificity, although recall and F1 scores slightly decreased. **Conclusions:** The results show that the modified Mask R-CNN model can detect gallstones with up to 0.89 accuracy, supporting the clinical applicability of the proposed method.

## 1. Introduction

Gallstones (cholelithiasis) are among the most common gastrointestinal disorders and may lead to serious complications. Although approximately 10–20% of the world population is affected, females have been identified as a significant risk group for gallstone disease (female-to-male ratio, ≈2:1) [1]. Several parameters can increase the prevalence, including age, diet, and hormonal fluctuations. In addition, obesity, family history, rapid weight loss, chronic hemolysis, liver cirrhosis, intestinal malabsorption, infections, and bile stasis have all been reported as factors that elevate the risk of gallstone formation [2]. The diversity and abundance of these factors make gallstones one of the most widespread gastrointestinal disorders. In this context, early and accurate diagnosis plays a critical role in effective management.

Magnetic resonance imaging (MRI) is not the primary modality for gallstone detection. Its sensitivity and specificity may be lower than those of conventional methods, such as ultrasonography and computed tomography (CT), in certain patient groups. However, MRI still plays an essential role in evaluating complications, including acute cholecystitis, pancreatitis, and biliary obstruction [3].

Considering the high prevalence of gallstone disease and the limited number of studies employing MRI for its evaluation, this study was designed to assess cholelithiasis using MRI. Furthermore, the visibility of cholesterol gallstones on computed tomography (CT) is relatively low, and ultrasonography performance may decrease in obese patients, emphasising MRI’s potential as a complementary imaging modality.

The time and human resources required for an efficient MRI assessment should also be considered. Accordingly, computer-aided support systems that can reduce the resources required for MRI-based diagnosis may ease the management of the condition. Recent image processing and deep learning techniques offer automated regional classification and accurate segmentation, enabling the development of an MRI-based computer-aided system.

Deep learning enables computers to learn from the most significant features automatically and directly from raw data, making this architecture particularly useful for digital spatial data, such as magnetic resonance images. At that point, Convolutional Neural Networks (CNNs) are specialised deep learning implementations for preserving and learning spatial relationships [4]. Accordingly, machine learning, particularly convolutional neural networks, has shown strong performance in analysing large medical image datasets and achieved higher accuracy than traditional radiological methods [5]. In an exemplary study, the authors incorporated deep convolutional neural networks with conventional machine learning methods to extract and classify disease regions in gastrointestinal tract images obtained from wireless capsule endoscopy [6]. In one ultrasound-based study, a custom CNN architecture was implemented using a modified generative adversarial network and a hybrid explanation framework to improve cholelithiasis classification performance, with detailed visual interpretability [7]. Moreover, in another recent study, a domain-adaptive deep learning model based on multi-scale feature extraction has been proposed for grayscale ultrasound images of the gallbladder. Authors have reported 99.63% accuracy and 99.50% F1 score across nine disease classes [8]. Similarly, another study implemented a machine learning–based automated categorisation system for the identification of choledocholithiasis. The study used a 3D convolutional neural network trained on data from 500 subjects. The model reportedly performed comparably to radiologists and maintained its accuracy across internal and external test sets [9]. A fully automated 3D gallbladder segmentation framework for MRCP data was also introduced in a recent study. The implemented mechanism combines shape-based features with a level-set refinement step. The reported mean Dice scores were above 0.90, underscoring that MRI-derived volumetric analysis can be performed reliably in a fully automated manner [10]. In another study, the YOLOv3 architecture was preferred to identify cholelithiasis and classify gallstones on CT images, with average accuracies of 92.7% for granular gallstones and 80.3% for muddy gallstones [11]. In a similar study, the classification of gallstone contents was investigated by applying a DCNN to SECT images (40 with and 30 without cholesterol stones) [12].

Recent advances in deep learning (DL) have enabled the automated diagnosis of gallbladder diseases using medical imaging. However, most studies focus on ultrasonography (US) and computed tomography (CT). Obaid et al. demonstrated that DL-based models can classify multiple gallbladder disease types, highlighting the diagnostic potential of artificial intelligence across diverse pathologies [13]. In another study, researchers systematically compared machine learning and deep learning approaches, noting that most AI-based solutions rely on US and CT, with limited attention to MRI data. They proposed an end-to-end DL framework for automatic gallbladder detection to reduce selection bias introduced by manually selected image slices [14]. Hasan et al. introduced an anatomy-aware DL model for gallbladder cancer classification using ultrasound images [15]. Similarly, Xiang et al. developed a contrast-enhanced CT-based DL model for differentiating malignant and benign gallbladder lesions [16]. Overall, while DL-based approaches for gallbladder disease diagnosis are expanding, MRI-based and cholelithiasis-focused decision support systems remain limited.

Although the gallbladder region can be clearly visualised on a routine MRI, the limited number of MRI-based deep learning studies on cholelithiasis may originate from practical challenges related to handling volumetric data and performing slice-wise annotation. The expert time and effort required to prepare well-annotated MRI datasets may explain the shift in focus from MRI to ultrasonography and computed tomography.

The previous literature has shown that integrating MRI with advanced image processing techniques may significantly improve diagnostic ability. Motivated by those studies, implementing a structured deep learning framework directly on MRI data appears to be a reasonable and promising approach for an efficient and functional supporting system. In this study, the aim was to develop an automated mechanism to assist in the assessment of cholelithiasis using T2-weighted axial MR images. For this purpose, an original dataset was created from the Radiology Archive of Izmir City Hospital to train a Mask R-CNN model with a ResNet-50 backbone. Overall, the proposed system is intended to assist with faster, more accurate diagnosis, particularly in cases that require urgent intervention and treatment.

Another contribution of this study is a graphical user interface (GUI), which was developed as part of the research framework to support the practical use of the proposed method. The interface is intended to facilitate application of the trained neural network models without requiring direct interaction with code or programming environments.

## 2. Materials and Methods

### 2.1. Original Dataset

An original dataset was created for this study, comprising anonymised T2-weighted axial MR images obtained from the Radiology Archive of Izmir City Hospital. The study was conducted in accordance with the Declaration of Helsinki and approved by the Institutional Review Board of Izmir City Hospital (approval number: 2025/431, Date: 10 September 2025). MRI examinations were performed using 1.5-T MR scanners (GE Optima360, Milwaukee, WI, USA). The imaging protocol included axial T2-weighted sequences acquired using half-Fourier acquisition single-shot turbo spin echo (HASTE/SSFSE). The imaging parameters were as follows: a field of view (FOV) of 420 × 380 mm, a section thickness of 4 mm with a 20% inter-slice gap, a matrix size of 272 × 320, a repetition time (TR) of 1200 ms, an echo time (TE) of 102 ms, and a scan duration of approximately 50 s.

Each image in the dataset corresponds to a different patient. For both cholelithiasis and normal subjects, the axial T2 slice that best represented the gallbladder in that examination was selected in order to construct a representative dataset. In cholelithiasis cases, stones were visible on the selected slice; however, the selection was performed to ensure appropriate case representation, rather than to maximise stone visibility. Therefore, no patient contributed more than one image to the dataset. The dataset was then randomly and blindly divided into training, validation, and test sets, which eliminates the risk of data leakage between splits. It should be noted that all ground truth annotations were created by a single radiologist (E. H.) with over 10 years of experience in abdominal imaging. Gallbladder boundaries and the presence of stones were annotated using a consistent visual protocol based on routine clinical assessment. It should be noted that this study does not propose a clinical segmentation tool; instead, it evaluates the feasibility of gallstone localisation on T2-weighted MR images. Because the gallbladder and stones are usually well-defined and visually distinct on the selected slice, expert radiologist annotations were considered sufficiently reliable for this technical investigation. Although no formal reproducibility analysis was performed, which may be a limitation, this is unlikely to significantly affect the conclusions. Future clinical studies should include assessments of inter- and intra-observer variability where appropriate.

**Figure 1:**
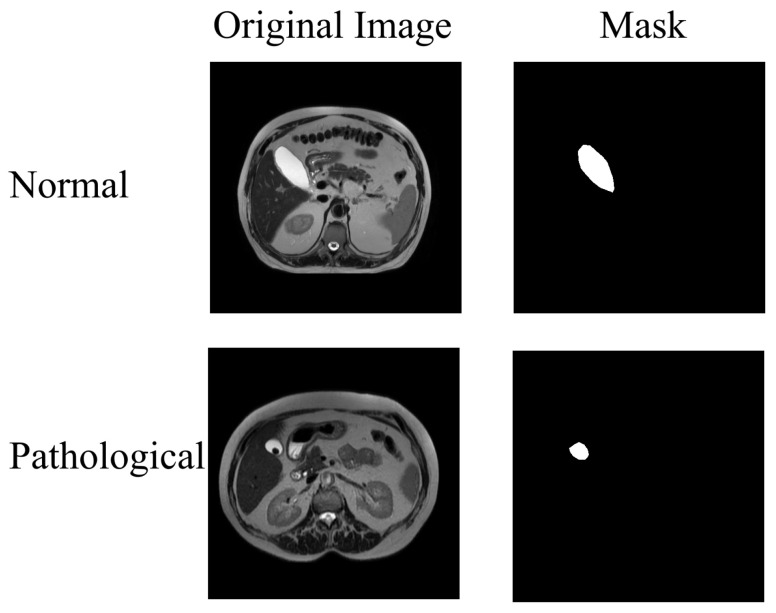
Example image–mask pairs from both normal and pathological cases in the original dataset.

During preprocessing, irrelevant elements such as patient information text and arms were manually masked. Subsequently, all images were resized to 512 × 512 pixels. Ground-truth masks were generated using a custom Python 3.1 interface, allowing an expert to delineate the region of interest. Binary masks were then produced from these manual annotations. Two example images and their corresponding masks from both pathological and normal cases are presented in Figure 1.

The original dataset consisted of 788 image-mask pairs, with 489 normal and 299 cholelithiasis-positive cases, for training and testing the model on normal and pathological gallbladder appearances.

### 2.2. Data Augmentation and Split

The original 788 pairs were first divided into training (70%), validation (15%), and testing (15%) subsets using a stratified random split to preserve the class proportions. Then, data augmentation was applied to the training samples before training to address class imbalance and improve model generalisation. Geometric transformations, including horizontal flip (*p* = 0.5), vertical flip (*p* = 0.2), small rotation (±10°, *p* = 0.6), and shift–scale–rotate (*p* = 0.5), were applied to both images and their corresponding masks to preserve spatial alignment. Appearance-based augmentations such as brightness/contrast adjustment (*p* = 0.5), gamma correction (*p* = 0.3), and motion blur (*p* = 0.2) were applied only to the images, while binary masks remained unchanged. Augmentations were implemented using the Albumentations library with a fixed random seed (42). A balancing strategy was used to reach a target of 1000 samples per class.

### 2.3. Implemented Model

In this study, a Mask Region-based Convolutional Neural Network (Mask R-CNN) model was adopted as the classifier. R-CNN models first make a rough estimation of object locations by proposing candidate regions and then classify these regions. Mask R-CNN extends this approach by adding the ability to produce a pixel-level mask for each detected region [17,18].

Accordingly, the main reason for selecting this model is its ability to define object boundaries while detecting the target regions within a single framework [19]. Producing pixel-level masks is particularly useful for MRI data, where structures often have irregular shapes and require accurate delineation. In addition, Mask R-CNN has shown stable performance in many medical imaging studies, making it a suitable choice for automated gallbladder analysis.

The proposed Mask R-CNN model was implemented with a ResNet-50 backbone as the feature extractor, together with a Feature Pyramid Network (FPN) architecture for better object representation at multiple scales [20]. This backbone configuration provides reliable feature extraction at different spatial resolutions, which is particularly important for medical image segmentation tasks. ResNet-50 was selected for its balanced trade-off between network depth and computational efficiency and is widely used in segmentation frameworks, including as the default backbone in Detectron2. Given the relatively small dataset, deeper architectures such as ResNet-101 or more complex models like EfficientNet were not preferred, as they may increase the risk of overfitting and lead to longer training times without a clear performance benefit in this setting.

In the study, the Detectron2 framework by Facebook AI was utilised for model implementation. The COCO instance segmentation protocol was used for training and testing. Accordingly, a ResNet-50 backbone with a Feature Pyramid Network (FPN), pretrained on the MS-COCO dataset, was used to transfer the model’s low-level feature representation capability. All layers of the network were fine-tuned during training. Stochastic gradient descent (SGD) with a base learning rate of 0.00025, momentum of 0.9, and a weight decay of 0.0001 was used during the training stage. The model was trained for 1000 iterations with a batch size of 2 using a single GPU. In addition, a validation-loss hook was incorporated to compute the validation loss at predetermined intervals without altering the training state. This mechanism provided the continuous monitoring of potential overfitting during optimisation.

The model was trained to perform two-class classification for the abnormality identification. For each test image, a stone-probability score was computed using the independent-event formulation:

$$p_{\mathrm{var}}=1-\prod_{i}\left(1-p_{i}\right),$$

where $p_{i}$ denotes the confidence of each instance predicted as a gallstone. This value served as the continuous classification score in the final reporting. Accordingly, the network aggregated the confidence scores of all the cases predicted as stone into a single image-level probability value using this formulation. This score was then used to determine the final binary decision. This approach enabled the implemented system to simultaneously localise the gallbladder region and classify stone images within the same framework.

It was observed that many MRI slices have a small gallbladder-like spine region. To prevent the module from focusing on visually similar but smaller incorrect regions, a post-processing step was also applied to improve diagnostic performance. Accordingly, all predicted instances were filtered using a confidence threshold of 0.30, and in cases where multiple candidate regions were detected, only the largest connected component was selected. Accordingly, small spurious detections, typically originating from nearby anatomical structures, did not influence image-level classification.

### 2.4. Squeeze-and-Excitation Modification

Each feature channel captures a distinct visual pattern in the image and corresponds to a parallel feature map produced by a convolutional layer. Based on this, a channel attention module learns the relative importance of the channels. It assesses which channels are more informative and increases their influence while suppressing less relevant ones [21].

Squeeze-and-Excitation (SE) blocks serve as lightweight channel attention modules. Their primary purpose is to emphasise more informative channel-wise features while suppressing less important ones [22]. In this mechanism, the spatial information of each feature map is first summarised via a global pooling step (squeeze), followed by recalibrating channel responses via learning importance weights (excitation) [23]. This approach often improves performance with a minimal computational cost. Accordingly, in addition to the standard Mask R-CNN architecture, a lightweight channel-attention mechanism was examined to evaluate whether its inclusion could improve the backbone.

The SE-augmented model was evaluated under the same conditions as the baseline model, including identical data splits, preprocessing steps, learning rate schedule, iteration count, anchor settings, and ROI (Region of Interest) head configuration. No additional data augmentation or optimisation adjustments were applied. Both the baseline and the SE-augmented models used the same post-processing stage. They were evaluated with the same metrics to ensure a fair comparison and clearly observe the effect of the SE modification.

### 2.5. Graphical User Interface

A graphical user interface (GUI) was also designed and implemented for clinical testing. The aim was to provide a simple frontend that allows expert radiologists to load and examine MRI slices quickly and easily. The proposed GUI displays the results directly on the original image and can be used without any technical background or coding skills.

The GUI was developed in the Python environment using Tkinter 8.6.12 for the interface and PyTorch 2.5.1 for model inference, with supporting libraries such as Pillow for image handling, Albumentations for preprocessing, segmentation-models-pytorch for the U-Net architecture, and OpenCV for post-processing and mask visualisation. In addition, a “requirements.txt” file was created to specify the necessary libraries. The GUI can be launched from a single “.bat” file that automatically checks for and installs the required packages before running the main script and opening the interface window.

**Figure 2:**
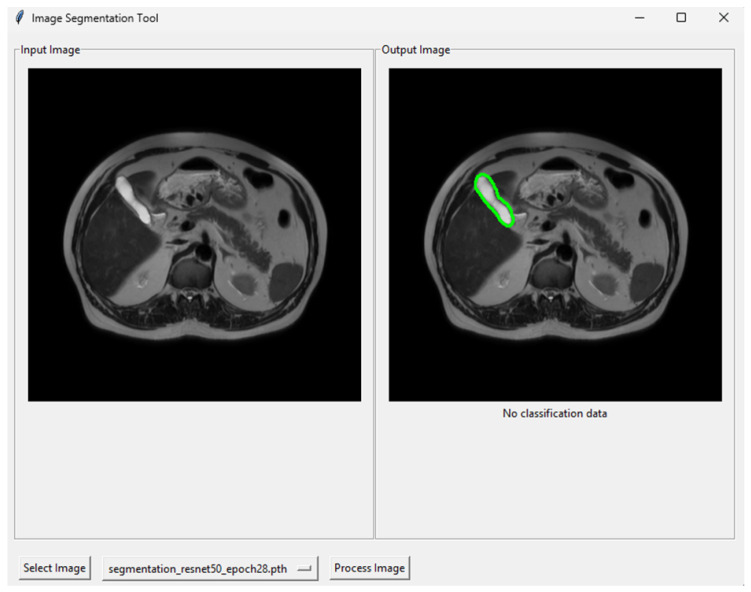
The implemented interface displays the selected MRI slice (**left**), and the segmentation result overlaid on the original image (**right**). The classification output is shown below the processed image when available.

The graphical interface provides three main functions: selecting the input image, choosing the model, and processing the image. The system pre-processes the selected image (resizing to 512 × 512), displays it in the left panel, feeds it into the chosen model for prediction, extracts the mask contours, overlays them on the original image, and shows the final result in the right panel. If a classification result, such as stone detection or a healthy state, is available, it is displayed at the bottom of the output image. An example view of the implemented interface is shown in Figure 2.

## 3. Results

The performance of the proposed two-class Mask R-CNN model was evaluated on the independent test set containing both stone and clean gallbladder images. For segmentation performance, Dice similarity coefficients were calculated by comparing the ground-truth binary masks with the model-generated masks of the largest connected component. To investigate the effect of the SE modification, the segmentation performance of the modified model was evaluated using the same test protocol. The comparison revealed a slight overall improvement in Dice coefficients, particularly in the abnormal (cholelithiasis) group, with increased mean and median scores and reduced variability. Moreover, a U-Net model with a ResNet-50 encoder backbone pre-trained on the ImageNet dataset was implemented in the study for segmentation performance comparison. U-Net is one of the most widely implemented convolutional neural networks, which is capable of more precise segmentations with fewer training samples [24]. The model was trained for 200 epochs with a batch size of 4 using the AdamW optimiser with a learning rate of 0.0001 and a weight decay of $10^{-8}$. A OneCycleLR scheduler was employed to manage the learning rate during training. To reduce overfitting and improve generalisation, the same dataset with augmented samples was used, and standard ImageNet normalisation was applied as an additional preprocessing step. Focal Loss with alpha = 0.8 and gamma = 2 was used, which is well suited for segmentation tasks with potential imbalance between foreground and background pixels. The training process was stopped at epoch 28, which yielded the highest validation Dice score, and was selected for evaluation. The model’s performance was tested on the same independent test set (unseen during training), and final predicted masks were generated using a sigmoid activation threshold of 0.5. Dice score metrics for the overall segmentation performance of all evaluated models are presented in Table 1, separately for normal samples and samples with stones.

Image-level stone detection performance was assessed using the predefined yellow-box criterion, which labels an image as “stone-positive” when the largest predicted instance belongs to the stone class and its confidence score exceeds the probability threshold. Based on this decision rule, accuracy, precision, recall, specificity, F1-score, and ROC-AUC were computed over all test samples. To assess the impact of the SE inclusion, the same evaluation strategy was applied to the updated model. Accordingly, a shift towards conservative classification was observed. While the model achieved higher precision and specificity, the lower recall led to decreased F1-score and ROC-AUC values. Therefore, it is not possible to conclude that overall classification superiority exists, despite a different balance between false positives and false negatives. Numerical results are presented in Table 2.

A confusion matrix (Table 3) was also generated to summarise the distribution of true positives, false positives, true negatives, and false negatives.

Bootstrapping (resampling with replacement 1000 times) was performed to estimate the stability of the presented modified and baseline model results. For each metric, 95% confidence intervals (CIs) were calculated on the held-out test set. The baseline model achieved: Accuracy = 0.818 (CI: 0.750–0.883), F1-score = 0.750 (CI: 0.643–0.844), ROC-AUC = 0.825 (CI: 0.750–0.897), and Dice = 0.869 (CI: 0.835–0.897). For the modified model, the results were: Accuracy = 0.808 (CI: 0.733–0.875), F1-score = 0.691 (CI: 0.568–0.800), ROC-AUC = 0.774 (CI: 0.701–0.848), and Dice = 0.895 (CI: 0.870–0.914). These findings show that both models achieved stable performance, and the precision–recall trade-off introduced by the SE modification is also reflected in the confidence ranges.

**Figure 3:**
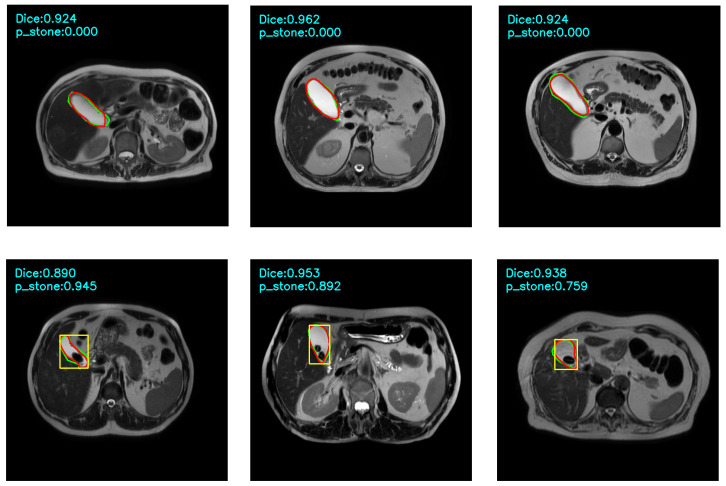
Visual results of the implemented classifier are shown. Red contours indicate the ground truth annotations, while green contours show the predicted segmentation results. A yellow bounding box is displayed if a gallstone is detected based on the classification criteria.

In addition to numerical evaluations, visual overlays were produced for qualitative assessment. For each test image, the predicted mask, ground-truth contour, and dice score were displayed in the output visualisation. When the yellow-box criterion was satisfied, a yellow bounding box was shown around the predicted region to indicate a positive stone detection. Example visual outputs are presented in Figure 3.

## 4. Discussion

This study was not intended to develop a fully automated screening system for complete MRI volumes. Instead, the proposed model serves as an expert-guided, slice-level decision-support tool to assist radiologists during routine navigation of axial T2 images. In clinical practice, diagnostic reasoning typically relies on a limited number of representative slices where suspicious findings are visually prominent, rather than on uniform analysis of all slices. Therefore, the model was trained and tested using single MRI slices that radiologists typically examine during routine clinical evaluation. The index slice was selected to best represent each case based on gallbladder visibility, not to maximise stone visibility. This approach simplifies the input space and facilitates integration into existing workflows, but it also introduces selection bias due to retrospective slice selection. Consequently, the reported performance should be regarded as evidence of feasibility for slice-level support under expert-guided conditions, rather than as a measure of end-to-end clinical performance. Future research should expand this framework to include prospectively defined slice-selection strategies or multi-slice evaluations to better assess robustness and generalisability, especially in contexts requiring automation beyond targeted expert interaction.

Ultrasound remains the primary modality for gallstone detection; however, magnetic resonance imaging (MRI) is frequently employed when complications are suspected or findings are inconclusive. In these scenarios, the proposed system can assist radiologists by automatically identifying the gallbladder and potential stones on T2-weighted images. This system is designed as an experimental decision-support prototype to complement, rather than replace, routine imaging when MRI is already part of the clinical workflow. Given the high prevalence of gallstones and the limited number of MRI-based studies, the present study investigates the application of MRI for cholelithiasis detection. In clinical practice, computed tomography has reduced sensitivity for cholesterol stones and exposes patients to ionising radiation. Furthermore, ultrasonography may yield suboptimal results in obese patients, making MRI a valuable alternative in selected cases.

In clinical settings, false negatives can lead to undetected gallstones, potentially delaying treatment and increasing the risk of complications. Although the SE-modified model demonstrated improved segmentation quality, it showed a slight decrease in recall and F1 Score. This trade-off indicates that enhanced localisation may reduce sensitivity. Consequently, the model’s performance should be interpreted with caution, particularly in high-risk diagnostic applications. Future research should investigate strategies to balance these metrics better, such as adjusting decision thresholds or integrating multiple decision criteria. Due to class imbalance in the dataset, accuracy alone may not adequately represent model performance. Therefore, F1-score and ROC-AUC were also considered essential for evaluating the impact of the observed precision–recall trade-off.

The inclusion of the SE module improved precision and specificity but reduced recall. This outcome may be attributed to the attention mechanism prioritising clear, strong features while suppressing ambiguous regions, resulting in a more conservative model. Such conservatism may be advantageous in supportive applications. In scenarios where missing a stone is critical, adjusting SE block parameters or employing adaptive thresholds could better balance precision and recall. A recent study evaluated gallbladder pathologies using ultrasonography by incorporating an SE module similar to the approach proposed here [25]. The reported results demonstrate significant potential.

From an operational perspective, the proposed system should be regarded as a second-reader tool, rather than a triage or screening solution. The system is designed to be applied after a radiologist has visually identified a potentially suspicious gallbladder region on a given slice, at which point the model provides localisation and confidence cues. In this context, the observed trade-off favouring higher specificity and precision at the expense of sensitivity may be acceptable in a decision-support context since false positives may unnecessarily distract the reader. In contrast, false negatives do not override the radiologist’s primary judgment. Therefore, operating thresholds were selected to prioritise conservative alerts consistent with a decision-support function. It should be noted that the present study does not evaluate clinical outcomes, reading time, or inter-observer agreement; therefore, no claims are made regarding direct clinical impact.

In the segmentation analysis, the baseline model achieved consistent dice scores for both normal and cholelithiasis cases. The modified model demonstrated a slight but distinct improvement, particularly within the abnormal group. The mean dice value increased from 0.8479 to 0.8934, while the standard deviation decreased substantially, indicating more homogeneous predictions in cases where gallstones may distort the gallbladder boundary. Normal samples also showed a modest improvement. These findings indicate that the architectural modification primarily enhanced model stability, rather than causing substantial changes in average performance.

In the experimental setup, the yellow-box rule was applied to all test images to retain only the largest predicted segment per image. This size-based filtering was intended to reduce false positives by preserving the most confident and spatially dominant region. Although this approach may decrease recall in cases involving small or multiple stones, it proved effective for a substantial portion of the test set. All thresholds, including confidence and post-processing parameters, were empirically optimised on the validation set and remained fixed during testing. In this study, a fixed threshold of 0.30 was empirically selected using early validation set results. While the threshold was not formally optimised, it achieved a reasonable balance between sensitivity and specificity. Future work will explore implementing an adaptive decision rule to enhance the classification system’s robustness. While a comprehensive ROC or PR curve analysis was not performed, the influence of this rule is apparent in the observed precision–recall trade-off.

The image-level results demonstrated a distinct trade-off. Under the yellow-box rule, the baseline model exhibited balanced performance, while the modified model adopted a more conservative approach. Increases in precision and specificity reduced false alarms and enhanced the reliability of positive predictions. However, this improvement was accompanied by a higher false-negative rate, potentially restricting the modified model’s applicability in clinical contexts where sensitivity is essential. Consequently, the modified model may be better suited for supportive functions, rather than as a primary diagnostic tool. Additionally, the dataset used in this study was derived from a single centre and had a relatively small sample size, limiting the generalisability of the findings. Future research should expand the dataset to include multi-centre and multi-device sources to improve model robustness and facilitate broader validation.

Compared to the U-Net, the Mask R-CNN model requires more computational resources due to its region proposal and instance segmentation architecture. Nevertheless, Mask R-CNN achieves more precise localisation, which can be essential in specific diagnostic contexts. Conversely, U-Net demonstrates faster performance and reduced computational demands, making it suitable for real-time applications or environments with limited resources. The graphical user interface (GUI) was evaluated using both models. Mask R-CNN yielded superior segmentation in pathological cases, whereas U-Net exhibited faster response times and lower memory usage. This trade-off should be evaluated in the context of the clinical setting and hardware constraints. The comparison with U-Net was conducted solely to illustrate segmentation performance. Differences in architecture and training were recognised, and no assertions regarding overall system superiority were made.

To evaluate the reliability of the results, a bootstrap-based analysis was conducted separately for both the baseline and modified models using 1000 resamples from the test set. The resulting 95% confidence intervals for Accuracy, F1-score, ROC-AUC, and Dice indicate that the reported values are stable and unlikely to be attributed to random variation. These intervals further support the model’s consistency and robustness. A predetermined number of training iterations was used to assess the feasibility of the proposed architecture. Continuous monitoring of the validation loss showed a consistent decrease, with stabilisation within the first 1000 iterations, indicating effective convergence. These results imply that the model can achieve robust performance with limited tuning. Future studies may explore longer training durations and hyperparameter optimisation to enhance performance further.

A qualitative inspection of the outputs confirmed that both models produced anatomically plausible segmentations. The visual overlay of ground-truth and predicted masks demonstrated consistent boundary alignment. Additionally, the yellow-box annotation increased the transparency of image-level decisions for end users.

A detailed analysis of the test results indicated that segmentation failures occurred in several challenging cases. In abnormal cases, false negatives were particularly evident when the gallbladder contained multiple small stones or when stones exhibited low contrast. In normal cases, some failures (such as Dice = 0) were due to the model incorrectly labelling other bright anatomical structures, rather than the gallbladder. These observations highlight the difficulty of addressing edge cases within a randomly selected test set. While the largest component rule reduced false positives, it occasionally discarded small valid regions. Future work could explore more adaptive strategies.

Overall, the study demonstrates that instance segmentation is a viable strategy for the automated analysis of gallbladder MR images. The modified architecture provided more stable segmentation and higher precision in cholelithiasis detection, although at the cost of reduced sensitivity. The model generated at least one predicted mask for every test image. However, in a subset of stone-positive cases, the predicted regions were not identified as positive according to the yellow-box decision rule. Specifically, 20 out of 46 stone-positive images (43.5%) were not marked with a yellow box, despite displaying visually reasonable segmentations. In these instances, the largest connected component was either classified as non-stone or did not surpass the predefined probability threshold. This outcome appears to result from the conservative decision strategy implemented, rather than a failure in segmentation. Although the largest-connected-component rule effectively suppresses false positives, it may reduce sensitivity in cases involving partial overlap, fragmented stone regions, or predictions with lower confidence. Future research could explore adaptive thresholds or multi-instance aggregation strategies to achieve a better balance between sensitivity and specificity.

The graphical user interface (GUI) was developed as a component of the research infrastructure, rather than as a clinically deployed tool. Nevertheless, the results indicate that, even in its current basic form, the interface may improve the usability of the proposed method in practical applications. It should be noted that the integration of the developed interface into the clinical workflow has not yet been evaluated.

This study faced several limitations. The absence of validation with multicentre datasets is a significant constraint. While precision and sensitivity improved, there was a slight increase in false-negative rates, potentially leading to missed cases of cholelithiasis. Additionally, the models were trained solely on 2D T2-weighted MR images, and their performance has not been assessed on other imaging sequences or volumetric data.

Future research should address these limitations by experimenting with alternative attention modules, integrating multi-sequence MR data, and optimising the decision threshold to balance sensitivity and specificity according to clinical requirements. Additionally, studies focusing on stone classification and gallbladder wall thickness measurement are recommended.

Recent studies in various domains have employed transformers and hierarchical training strategies to enhance segmentation or classification performance under weak supervision or limited data. For instance, synthetic data generation and progressive feature learning have been utilised in pavement crack detection and sonar image analysis, respectively [26,27]. Although these applications differ in scope, they demonstrate the potential of advanced learning strategies for tasks that demand large training sets to address complex classification challenges, thereby informing possible future enhancements to the present model.

Specialised AI applications in medical imaging are increasingly influencing clinical practice and are becoming more integrated into clinical workflows. However, it should not be forgotten that this technological innovation, which is still in its early stages, also carries limitations and risks [28]. Weak generalisation in the applied models, the potential for memorisation (overfitting), and deficiencies in the representation of the training data require that AI continue to be used carefully in clinical applications and under continued expert supervision. Therefore, despite the diagnostic and assistive potential demonstrated in this study, DL models should be regarded not as independent, standalone decision-makers, but as supportive tools.

## 5. Conclusions

A deep learning–based automated classification and localisation mechanism was implemented in the study. One of the primary goals was to provide a supporting detection system for gallbladders on MRI data. Accordingly, several up-to-date models were trained and tested with an original dataset. The proposed two-class Mask R-CNN model successfully segmented the gallbladder and detected cholelithiasis with high accuracy in T2-weighted MR images. Moreover, the modified architecture improved segmentation consistency, especially in pathological cases, as evidenced by higher Dice scores and reduced variability. Classification metrics showed a shift toward higher precision and specificity at the cost of reduced sensitivity. This trade-off suggests that the modified model may be more suitable for screening-oriented or decision support scenarios where minimising false-positive findings is desirable. Overall, these findings indicate that the proposed pipeline with a graphical user interface may facilitate gallbladder detection and support future clinical applications of deep learning–based gallbladder analysis; however, further prospective and multi-centre validation is required before routine clinical implementation.

## Figures and Tables

**Table 1:** Dice statistics for normal samples and samples with stones.

Normal Samples (n = 74)					
**Model**	**mean**	**std**	**median**	**min**	**max**
Baseline Mask R-CNN	0.8812	0.1567	0.9211	0.0000	0.9667
Modified Mask R-CNN	0.8946	0.1554	0.9297	0.0000	0.9679
Baseline U-Net	0.9159	0.0515	0.9331	0.6394	0.9705
**Samples with Stones (n = 46)**					
**Model**	**mean**	**std**	**median**	**min**	**max**
Baseline Mask R-CNN	0.8479	0.2106	0.9249	0.0000	0.9656
Modified Mask R-CNN	0.8934	0.0585	0.9047	0.7108	0.9569
Baseline U-Net	0.8756	0.1639	0.9193	0.0000	0.9701

**Table 2:** Comparison of image-level classification metrics between baseline and modified Mask R-CNN models.

Model	Accuracy	Precision	Recall (Sensitivity)	Specificity	F1	ROC-AUC
Baseline	0.8167	0.7857	0.7174	0.8784	0.7500	0.8236
Modified	0.8083	0.8966	0.5652	0.9595	0.6933	0.7738

**Table 3:** Comparison of confusion matrices for baseline and modified Mask R-CNN models.

Model		Pred_Clean	Pred_Stone
Baseline	GT_clean	65	9
	GT_stone	13	33
Modified	GT_clean	71	3
	GT_stone	20	26

## Data Availability

The original MRI data used in this study are not publicly available due to institutional privacy policies. The code and data used for training and evaluation are available from the authors upon reasonable request.

## Abbreviations

The following abbreviations are used in this manuscript:

MRI	Magnetic Resonance Imaging
R-CNN	Region-based Convolutional Neural Network
CT	Computed Tomography
CNN	Convolutional Neural Networks
MRCP	Magnetic resonance cholangiopancreatography
YOLO	You Only Look Once
DCNN	Deep Convolutional Neural Network
SECT	Single Energy Computed Tomography
GUI	graphical user interface
COCO	Common Objects in Context
ResNet-50	Residual Networks (50 Layers)
FPN	Feature Pyramid Network
AI	Artificial Intelligence
MS-COCO	Microsoft Common Objects in Context
SGD	Stochastic Gradient Descent
SE	Squeeze-and-Excitation
ROI	Region of Interest
std	Standard Deviation
ROC-AUC	Area under the Receiver Operating Characteristic Curve
Pred	Predicted
GT	Ground Truth
